# Genetic variability and consequence of *Mycobacterium tuberculosis* lineage 3 in Kampala-Uganda

**DOI:** 10.1371/journal.pone.0221644

**Published:** 2019-09-09

**Authors:** Eddie M. Wampande, Peter Naniima, Ezekiel Mupere, David P. Kateete, LaShaunda L. Malone, Catherine M. Stein, Harriet Mayanja-Kizza, Sebastien Gagneux, W. Henry Boom, Moses L. Joloba

**Affiliations:** 1 Department of Medical Microbiology, College of Health Sciences, Makerere University, Kampala, Uganda; 2 Department of Veterinary Medicine, Clinical and Comparative medicine, College of Veterinary Medicine, Animal Resources and Bio Security, Makerere University, Kampala, Uganda; 3 Department of Pediatrics and Child Health College of Health Sciences, Makerere University, Kampala, Uganda; 4 Uganda-Case Western Reserve University Research Collaboration, Kampala, Uganda; 5 Tuberculosis Research Unit, School of Medicine, Case Western Reserve University and University Hospitals of Cleveland, Cleveland, OH, Uinted States of America; 6 Department of Population and Quantitative Health Sciences, School of Medicine, Case Western Reserve University, Cleveland, OH, Uinted States of America; 7 Swiss Tropical and Public Health Institute, Basel, Switzerland; 8 University of Basel, Basel, Switzerland; The University of Georgia, UNITED STATES

## Abstract

**Background:**

Limited data existed exclusively describing *Mycobacterium tuberculosis* lineage 3 (MTB-L3), sub-lineages, and clinical manifestations in Kampala, Uganda. This study sought to elucidate the circulating MTB-L3 sub-lineages and their corresponding clinical phenotypes.

**Method:**

A total of 141 *M*. *tuberculosis* isolates were identified as *M*. *tuberculosis* lineage 3 using Single nucleotide polymorphism (SNP) marker analysis method. To ascertain the sub-lineages/sub-strains within the *M*. *tuberculosis* lineage 3, the direct repeat (DR) loci for all the isolates was examined for sub-lineage specific signatures as described in the SITVIT2 database. The infecting sub-strains were matched with patients’ clinical and demographic characteristics to identify any possible association.

**Result:**

The data showed 3 sub-lineages circulating with CAS 1 Delhi accounting for 55% (77/141), followed by CAS 1-Kili 16% (22/141) and CAS 2/CAS 8% (12/141). Remaining isolates 21% (30/141) were unclassifiable. To explore whether the sub-lineages differ in their ability to cause increased severe disease, we used extent of lung involvement as a proxy for severe disease. Multivariable analysis showed no association between *M*. *tuberculosis* lineage 3 sub-lineages with severe disease. The risk factors associated with severe disease include having a positive smear (OR = 9.384; CI 95% = 2.603–33.835), HIV (OR = 0.316; CI 95% = 0.114–0.876), lymphadenitis (OR = 0. 171; CI 95% = 0.034–0.856) and a BCG scar (OR = 0.295; CI 95% = 0.102–0.854).

**Conclusion:**

In Kampala, Uganda, there are three sub-lineages of *M*. *tuberculosis* lineage 3 that cause disease of comparable severity with CAS-Dehli as the most prevalent. Having HIV, lymphadenitis, a BCG scar and a smear negative status is associated with reduced severe disease.

## Introduction

Seven major lineages of human-adapted *Mycobacterium tuberculosis complex* (MTBC) are preferentially distributed in specific geographical niches, where they are the primary cause of Tuberculosis (TB). Geographic dispersion includes *Mycobacterium tuberculosis* (*M*. *tuberculosis*) lineage 1 (Indo Oceanic) found in areas along the Indian ocean, *M*. *tuberculosis* lineage 2 found majorly in east Asia, *M*. *tuberculosis* lineage 3 found in East Africa and India, *M*. *tuberculosis* lineage 4 (Euro-American) found mainly in Africa, Europe and America, *M*. *tuberculosis* lineage 5 & 6 (*M*. *africanum1 &* 2) found exclusively in West Africa and *M*. *tuberculosis* lineage 7 found primarily in Ethiopia [1–3]. The *M*. *tuberculosis* lineage 3 (MTB-L3), also known as the Central Asian strains (CAS), occurs predominantly in areas around the Indian Ocean, East Africa and India [4, 5]. The genetic diversity of the CAS can be defined based on specific single nucleotide polymorphisms (SNPs) [6, 7], genomic deletion, also known as long sequence polymorphism (LSP) [4, 5], and a particular spoligotype pattern [8]. The latter can further subdivide the main *M*. *tuberculosis* lineage 3 into specific sub-lineages [8]. Emergence and spread of *M*. *tuberculosis* lineages to other niches (where they were originally absent) has been associated with immigration, clinical and demographic factors, as well as evolution of MTB strains [9, 10]. Understanding mechanisms shaping transmission of MTB strains can provide a lead about the potential approaches for TB control.

The data from our previous studies showed that in Kampala, Uganda, there are 3 main *M*. *tuberculosis* lineages circulating, of these 11% were *M*. *tuberculosis* lineage 3 [11]. Moreover, findings also revealed that all the *M*. *tuberculosis* predominant in Kampala were equally virulent (based on cavitation as a proxy for virulence). Nevertheless, elsewhere authors have reported that different *M*. *tuberculosis complex* lineages infections present with specific clinical phenotypes [3]. The failure to demonstrate specific clinical outcomes in our earlier dataset might be attributable to comparing genetically heterogeneous *M*. *tuberculosis complex* main lineages; this could have confounded our results thereby suggesting no difference in virulence. Differences in bacterial characteristics have provided insight into how the *M*. *tuberculosis complex* bacteria cause disease, and why some are geographically wide spread. For instance, the Beijing strains that belong to *M*. *tuberculosis* lineage 2 are highly virulent, prone to drug resistance and BCG vaccination is not protective. This may partly explain why they are a global threat [12–15]. Additionally, strains of *M*. *tuberculosis* lineage 4 are associated with pulmonary tuberculosis and severe lung consolidation, less virulent [16] and prone to anti-tuberculosis drug resistance [17] as opposed to other sub lineages. Similarly *Newton et al*,[18] showed that sub-lineages of *M*. *tuberculosis* lineage 3 cause severe disease; Stucki et al, [19] and Hershberg, 2016 [20] showed that *M*. *tuberculosis* lineage 5–7 have a narrow host range, thus they are restricted to particular geographical niche. Therefore, accurate understanding of *M*. *tuberculosis complex* sub-lineages and their clinical outcomes can bolster the development of appropriate intervention strategies that more effectively target the circulating strains.

Given that background in the current study, we are describing sub-lineages/sub-strains within the main *M*. *tuberculosis* lineage 3, the least dominant MTB lineage in kampala. To answer this question we shall start by analyzing the MTB direct repeat (DR) loci for sub lineages within M. tuberculosis lineage 3 as well as understanding the demographic and clinical manifestation of patients infected with MTB-L3 sub lineages. With such an approach, we can describe whether sub-lineages of *M*. *tuberculosis* lineage 3 prevalent in Kampala, Uganda differ in their ability to cause severe disease (extent of lung involvement abnormalities) as evaluated by chest x-ray.

## Materials and methods

### Study design and *M*. *tuberculosis* isolates

The *M*. *tuberculosis* isolates used in this study were obtained from adult (≥ 18 years) patients (index cases) and their household contacts (HHCs), confirmed with pulmonary TB by culture in a cross sectional study (2002–2012) in Kawempe division Kampala, Uganda [11, 21], where the data for the current study is coming from. The HHCs were TB patients who had stayed with an index patient for at least 7 consecutive days for the previous 3 months. The index cases residing with 1 or more HHCs were enrolled in the study through the clinic at the Uganda National TB and leprosy program at Mulago Hospital or by referral to the TB research clinic at Mulago Hospital or through public sensitization in Kawempe division. Adults with clinical signs (a positive chest x-ray or sputum smear positive) suggestive of tuberculosis provided a sputum sample for culture following standard laboratory procedures. The patients with active TB were treated using a short course therapy of Isoniazid (INH), rifampicin (RIF), pyrazinamide and ethambutol for 2 months, followed by 4 months of INH and RIF. The cultured samples were later tested for drug resistance, patients with resistant MTB isolates were provided with treatment according to the TB program guidelines. The HHCs ≤ 5 years old, HIV and TST-positive were prophylactically treated with INH for 6–9 months. Patients’ baseline demographic and clinical variables such age, sex, HIV status, employment status, status on income, TB cavitation on chest x-ray (present or absent), ethnicity (Bantu & others), status of smoking, body mass index (BMI) calculated from height & weight, alcohol drinking, presence of BCG scar, whether patients have night sweats, knowledge of TB in the past, presenting with hemoptysis (cough with blood), having swollen lymph nodes (lymphadenitis), evaluation of extent of lung involvement on chest radiography (classified as normal, mild, moderate, or far advanced) and smear status (positive or negative), were recorded by a medical physician or a laboratory technician.

### Genomic DNA extraction and genotyping *M*. *tuberculosis* isolates

DNA extraction for 141 *M*. *tuberculosis* isolates and SNP (lineage-specific SNP for *M*. *tuberculosis* lineage 3: Rv0129c_0472n) typing to identify *M*. *tuberculosis* lineage 3 was performed as described by Wampande *et al*, [11]. To determine the sub-lineages of *M*. *tuberculosis* lineages 3, the isolates were further analyzed with a spoligotyping commercial kit as described by Kamerbeek et al, [22], the shared international type (SIT) spoligotyping were assigned according to SITVIT and SITVIT2 database [8, 23].

### Statistical analysis

Baseline variables were given as means, median if continuous while the categorical variables were described in percentages. The outcome of our analysis was a patient with minimal (lung infiltrates of slight to moderate density and disease present to a small portion of one or both lungs with no cavitation) or advanced disease (lesions more extensive than minimal disease with cavitation) on chest x-ray examination [24]. Univariate analysis was perfomed and the chi square test or Fisher’s exact test was used to compare the distribution of categorical variable by disease. Variables in univariate analyis with *P ≤* 0. 2; except HIV a known risk factor for TB, were included in the multivariable logistic model. Multivariable logistic regression was used to evaluate the association between sub-lineages (sub strains) of *M*. *tuberculosis* lineage 3 (independent variable) and extent of lung involvement (minimal or advanced) disease on chest x-ray (dependent variable). The 2 individuals infected with CAS were excluded from the analysis because of the small number. Age, sex, smear status, HIV status, BCG scar, smoking status, swollen lymph nodes (lymphadenitis) and BMI were used as adjusters. All analyses were conducted with Stata software, version 12 (StataCorp, College Station, Texas).

### Ethics

The institutional review boards and ethics committees at University Hospitals of Cleveland, Makerere University, and the National HIV/AIDS Research Committee as well as the Uganda National Council for Science and Technology approved the study protocols. All patients gave written informed consent for study participation, including pre- and post- HIV test counseling.

## Results

**Fig 1:**
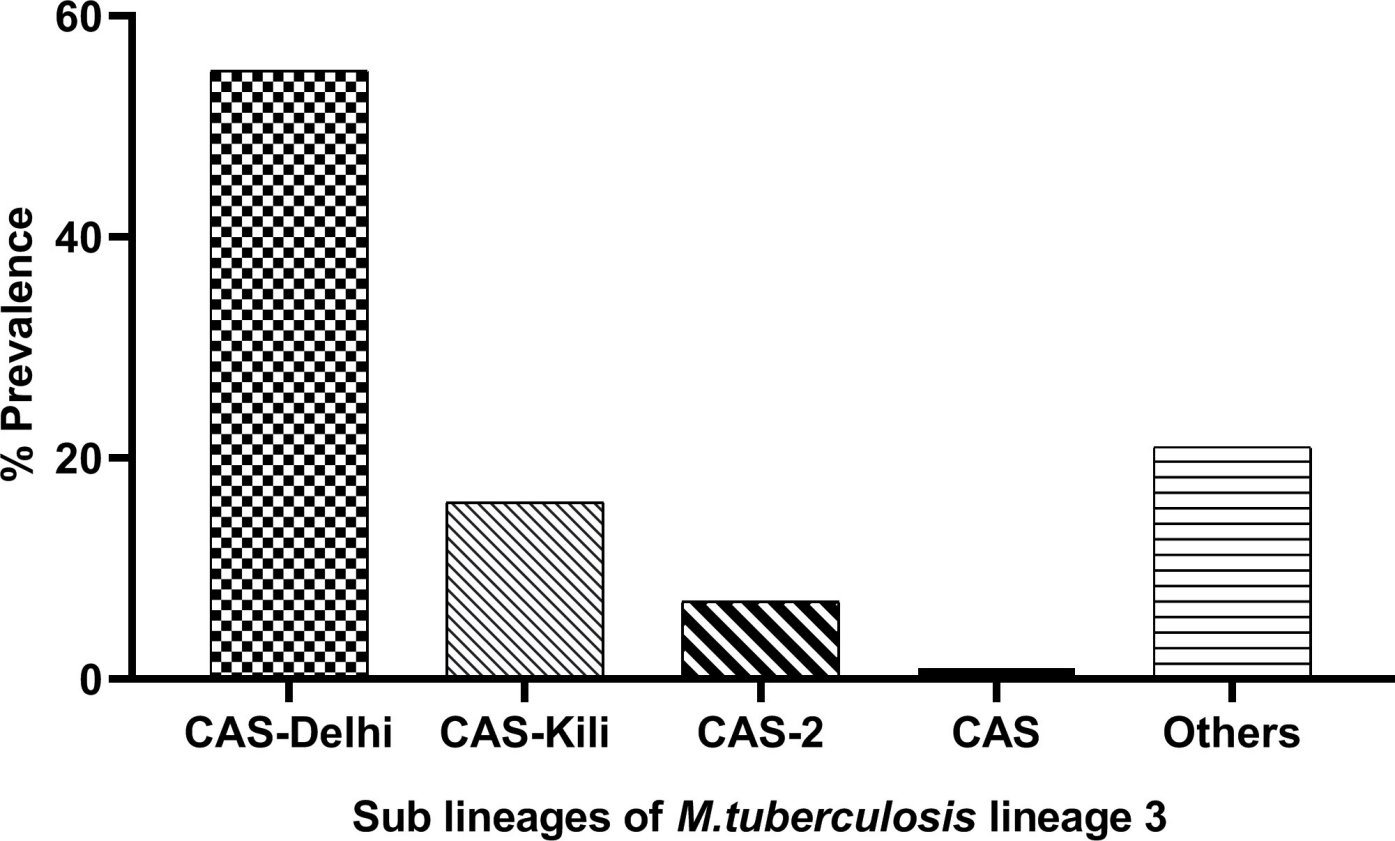
Sub-lineages of *M*. *tuberculosis* lineage 3. The sub-lineages were identified by spoligotyping as described in Materials and Methods, N = 141.

In the parent study we genotyped 1286 isolates of these 11% (141/1286) were MTB lineage 3. Of the 141 patients with pulmonary tuberculosis and infected with *M*. *tuberculosis* lineages 3, 77 (55%) were infected with CAS 1-Dehli, 22 (16%) were infected with CAS 1-Kili, 10 (7%) were infected with CAS 2, 2 (1%) were infected with CAS and the rest 30 (21%) were infected with *M*. *tuberculosis* lineage 3 sub lineages not yet defined in the SIT/VIT2 spoligotype database [8] (Fig 1 & S1 Table). The most frequent SITs were SIT26 30% (43/141) followed by SIT21 16% (23/141), SIT25 11% (16/141), while the rest were ≤ 7%, those considered as orphans were 12% (17/141) (S1 Table and S2 Table).

### Demographic and clinical characteristics of the study participants

For the analysis we included 141 *M*. *tuberculosis* lineage 3 isolates, each corresponding to a tuberculosis patient.

The description of the patients demographic and clinical characteristics has been detailed in Table 1; the proportions of the patients’ characteristics for the different variables among the sub-lineages of *M*. *tuberculosis* lineages 3 (Table 1) were generally similar irrespective of the MTB sub-lineage. From now onwards we have excluded the CAS strains in the analysis due to a small number (2 strains).

**Table 1:** Participant characteristics infected with different *M. tuberculosis* sub lineages.

	Variable	CAS-Dehli n (%)	CAS-Kili n (%)	CAS-2 n (%)	Others n (%)
Sex	Male	43 (56)	12 (55)	5 (50)	10 (33)
	Female	34 (44)	10 (45)	5 (50)	20 (67)
Age*	< 30 years	54 (70)	12(55)	7(70)	20(67)
	≥ 30 years	23 (30)	10(45)	3(30)	10(33)
Smear status^#^	Positive	59 (76)	15 (68)	7 (70)	17 (57)
	Negative	15 (19)	7 (32)	3 (30)	11(37)
	ND	3 (5)	0	0	2 (6)
Extent of lung involvement	Minimal disease	40 (51)	11(50)	3 (30)	15 (50)
	Advanced disease	37 (49)	11 (50)	7 (70)	15 (50)
HIV status^#^	Positive	29 (37)	10 (45)	5 (50)	15 (50)
	Negative	41(53)	12 (55)	5 (50)	13 (43)
	ND	7 (10)	0	0	2 (7)
BCG scar^#^	Present	45 (58)	10 (45)	2 (20)	14 (47)
	Absent	24 (31)	10 (45)	5 (50)	12 (40)
	ND	8 (12)	2 (10)	3 (30)	4 (13)
Cavity^#^	Present	34 (44)	12 (55)	7 (70)	15 (50)
	Absent	34 (44)	5 (22)	2 (20)	11(37)
	ND	9 (12)	5 (22)	1 (10)	4 (13)
Smoking status^#^	Never smoked	46 (60)	15(68)	8(80)	21 (70)
	Ever smoked	27 (35)	6 (27)	2(20)	7(23)
	ND	4 (5)	1(5)	0	2(7)
Drinking alcohol^#^	Yes	20 (26)	6(27)	2(20)	5(17)
	No	55 (70)	15(68)	8(80)	23(77)
	ND	2 (4)	1(5)	0	2(6)
Tribe^#^	Ganda	54 (69)	17(77)	6 (60)	20(67)
	Non-Ganda	21(27)	4(23)	4(40)	8(27)
	ND	2 (4)	0	0	2(6)
Coughing^#^	Cough blood	12 (15)	2(9)	1(10)	3(10)
	No blood	63 (81)	20 (91)	9(90)	27(90)
	ND	2 (4)	0	0	0
Fever	Yes	48 (62)	16(73)	5(50)	19(63)
	No	29 (37)	6(27)	5(50)	11(37)
Night sweat^#^	Yes	51(65)	18(82)	3(30)	17(57)
	No	26(33)	4(18)	7(70)	12(40)
	ND	0(0)	0	0	1(3)
Lymphadenitis^#^	Yes	5 (6)	4 (18)	0	3 (10)
	No	69 (89)	18 (82)	10 (100)	26 (87)
	ND	3 (5)	0	0	1(3)
BMI*	Under weight	39 (51)	10(45)	5(50)	14(47)
	Normal weight	38 (49)	12(55)	5(50)	16(53)
Employed^#^	Yes	8 (10)	3(14)	2(20)	4(13)
	No	12 (15)	3(14)	2(20)	6(20)
	ND	57 (74)	16(72)	6(60)	20(67)
Income^#^	Low	18 (23)	6(27)	3(30)	6(20)
	High	19 (24)	7(32)	3(30)	7(23)
	ND	40(53)	9(40)	4(40)	17(57)
TB in the past^#^	Yes	1 (1)	1(5)	1 (10)	1 (3)
	No	69 (88)	21(95)	9(90)	26(87)
	ND	7 (11)	0	0	3(10)

^#^ ND refers to not determined

*For age, mean = 27.43 years and median = 28 years: BMI mean = 18.86 kg/m^2^ and median = 18.61 kg/m^2^

### Risk factors associated with MTB lineage 3 infections

In all the analyses, CAS1-Dehli was used as the reference since is the most prevalent, and we set out to understand why it is dominant in comparison with other sub lineages circulating in the study area. Univariate analysis showed that disease severity (extent of lung involvement: minimal versus advanced disease) was not associated with any of the sub-lineages of *M*. *tuberculosis* lineage 3 (P≥ 0.05).

Risk factors such as sex (OR = 2.79; CI 95% = 1.408–5.564), smear status (OR = 4.35; CI 95% = 1.849–10.231), cavitary TB (OR = 11.667; CI 95% = 4.863–27.991), and smoking status (OR = 2.865; CI 95% = 1.331–6.16), were significantly associated with advanced severe disease. Presence of BCG scar was protective (OR = 0.326; CI 95% = 0.153–0.691). Others variables, for instance age, HIV status, alcohol drinking, tribe, coughing, fever, night sweats, BMI (under weight = <18.5 kg/m^2^, normal weight = ≥18.5–25 kg/m^2^), lymphadenitis, employment status, income and history of TB in the past are not associated with (P≥ 0.05) severe TB disease (Table 2).

**Table 2:** Univariate analysis for odds of developing severe disease based on extent of disease on chest x-ray.

	Proportion of patients with severe disease n (%)		*uOR	*uCI (95%)
MTB lineage 3 sub strains	38 (49)	CAS-Dehli	1	1
	11 (50)	CAS-Kili	1.05	0.41–2.71
	7 (70)	Cas	2.46	0.59–10.20
	15 (50)	Unknown lineage 3 strains	1.05	0.45–2.44
Age^1^	49 (53)	≤ 30 years	1	1
	21 (47)	>30 years	0.754	0.37–1.53
Sex^2^	26 (38)	Female	1	1
	44 (62)	Male	2.80	1.41–5.56
Smear status ^3^	9 (25)	Negative	1	1
	58 (59)	Positive	4.35	1.85–10.23
HIV status^4^	40 (56)	Negative	1	1
	27 (46)	Positive	0.65	0.33–1.31
BCG scar ^5^	34 (67)	Absent	1	1
	28 (39)	Present	0.33	0.15–0.69
Cavity ^6^	10 (19)	Absent	1	1
	50 (74)	Present	11.67	4.86–27.99
Smoking status^7^	37(41)	Never smoked	1	1
	24 (67)	Ever smoked	2.86	1.33–6.17
Drinking alcohol^8^	49(49)	No	1	1
	17 (52)	Yes	1.13	0.51–2.48
Tribe^9^	20 (53)	Non-ganda	1	1
	48 (49)	Ganda	0.88	0.42–1.87
Coughing^10^	60 (50)	No blood	1	1
	9 (50)	Cough blood	0.98	0.36–2.65
Fever^11^	25(49)	No	1	1
	45 (51)	Yes	1.11	0.56–2.22
Night sweat^12^	22(45)	No	1	1
	47 (53)	Yes	1.43	0.71–2.88
Lymphadenitis^13^	65 (53)	No	1	1
	3(25)	Yes	0.30	0.08–1.15
BMI^14^	39(57)	Under weight	1	1
	31(43)	Normal weight	0.58	0.29–1.13
Employed^15^	16(70)	No	1	1
	12 (70)	Yes	1.05	0.27–4.133
Income^16^	17(51)	High	1	1
	17 (47)	Low	0.84	0.33–2.17
TB in the past^17^	63(50)	No	1	1
	1 (25)	Yes	0.33	0.03–3.24

1 = no data missed

2 = no data missed

3 = 5 missed data for smear status

4 = 9 missed data for HIV status

5 = 17 missed data for BCG

6 = 20 missed data for cavity

7 = 7 missed data for smoking status

8 = 5 missed data for drinking alcohol

9 = 4 missed data for tribe

10 = 2 missed data for coughing with blood

11 = no data missed

12 = 1 missed data for night sweat

13 = 4 missed data for lymphadenitis

14 = no data missed

15 = 99 missed data for employment

16 = 70 missed data for income and

17 = 10 missed data for TB in the past

* u- Unadjusted OR and CI at 95% were obtained by logistic regression

### Multivariable analysis for association between severe lung disease and sub lineages of *M*. *tuberculosis* lineage 3

In the multivariate analysis after adjusting for sex, smear status, HIV status, BCG scar, smoking status and lymphadenitis, the data suggests that severity of TB disease is not dependent on the *M*. *tuberculosis* sub lineages (P ≥ 0.05).

Risk factors independently associated with disease severity included having a positive smear on sputum analysis (OR = 9.384; CI 95% = 2.603–33.835): HIV patients (OR = 0.316; CI 95% = 0.114–0.876), patients with lymphadenitis (OR = 0. 171; CI 95% = 0.034–0.856) and those with a BCG scar (OR = 0.295; CI 95% = 0.102–0.854) are less likely to have a severe TB disease (Table 3).

**Table 3:** Multivariable analysis for odds of developing severe disease.

		*aOR	*aCI (95%)
MTB lineage 3 sub strains	CAS-Dehli	1	1
	CAS-Kili	1.11	0.31–3.97
	CAS	5.88	0.36–95.76
	Unknown lineage 3 strains	1.69	0.49–5.85
Sex^1^	Female	1	1
	Male	2.233	0.82–6.09
Smear status^2^	Negative	1	1
	Positive	9.38	2.60–33.84
HIV status^3^	Negative	1	1
	Positive	0.32	0.11–0.88
BCG scar^4^	Absent	1	1
	Present	0.30	0.10–0.85
Smoking status^5^	Never smoked	1	1
	Ever smoked	2.45	0.84–7.20
Lymphadenitis^6^	No	1	1
	Yes	0.17	0.03–0.86

1 = no data missed

2 = 5 missed data for smear status

3 = 9 missed data for HIV status

4 = 17 missed data for BCG

5 = 7 missed data for smoking and

6 = 4 missed data lymphadenitis

* a- adjusted OR and CI at 95% obtained by logistic regression

## Discussion

*M*. *tuberculosis* infections are of global concern, therefore understanding the drivers of disease progress and spread is paramount. Host and environment factors have been suggested as key players among others that can bolster TB spread, there is also overwhelming evidence that bacterial diversity of *M*. *tuberculosis* may impact the dynamics of TB outcomes among those patients infected with the bacteria [16]. In the current study, we sought to determine whether sub-lineage variations within *M*. *tuberculosis* lineage 3 could influence disease severity outcome. Firstly, we characterized the sub-lineages within the main *M*. *tuberculosis* lineage 3 circulating in central Kampala. Secondly, we investigated for the clinical and epidemiological risk factors associated with sub-lineage infections. Such data is important in designing appropriate strategies for the management of TB.

In our study, among sub-lineages of *M*. *tuberculosis* lineage 3, the most successful sub-lineage was CAS 1-Dehli that causes at least 50% of the pulmonary TB, followed by CAS 1-Kili and CAS. This current data is contrary to earlier findings by Asiimwe et al, [25] in central Uganda, who showed that CAS 1-Kili was the most prevalent sub-strain, yet Bazira et al, [26] in western Uganda observed only CAS-Dehli sub-strains. In another study that exclusively considered extra pulmonary TB showed CAS 1-Dehli as the most prevalent, the previous 2 studies compares well with the current data [27]. Despite these incongruences, we argue our data is more robust since spoligotyping was performed on isolates that were first confirmed as *M*. *tuberculosis* lineage 3 by SNP [7] typing. The approach of defining first the main MTB lineage by SNP typing reduces on the errors of misclassifying intra lineage sub strains by spoligotyping since the direct repeat loci is prone to convergent evolution [6]. The other studies described exclusively used spoligotyping technique alone to define the sub lineages, and this could result in misclassification of sub lineages due to convergent evolution, thereby impacting the data. Moreover, in addition to MTB-L3 sub lineages, they considered other MTB lineages in the same study, which can disproportionately misrepresent the status quo due to overrepresentation of other sub lineages in the study area [11, 28]. Our current data demonstrated quite a number of isolates, 21% (30/141) that could not be classified in any of the known sub lineage. This finding leads one to consider that these might be unknown strains. Nevertheless, we cannot rule out the possibility of mixed (having more than one sub lineage) infections in patients as earlier reported by Dickman et al, [29] who studied isolates from the same study area. Such a scenario produces muddled finger prints which cannot be ascribed to any of the known shared international type (SIT) spoligotypes in the SITVIT2 database. Efforts are underway to fully characterize these supposedly “unknown strains” and have them undoubtedly described to the *M*. *tuberculosis* research community.

From our current data, to assess why CAS 1-Dehli is the most successful sub lineage in causing disease, we hypothesized that sub-lineages within *M*. *tuberculosis* lineage 3 differ in their ability of causing advanced severe disease; we defined severe disease as extent of lung engrossment with TB specific lesions and cavitation (minimal or advanced disease) on chest x-ray. Our data shows that the *M*. *tuberculosis* sub-lineages circulating in central Uganda equally cause disease in the infected patients (P ≥ 0.05). The CAS-sub-lineage suggests an association with severe disease (aOR = 5.9; aCI = 0.36–95.76), but then again due to the small sample size the wide confidence interval does not support the finding, this calls for another bigger study to substantiate on this observation. Contrary to our findings, *M*. *tuberculosis* lineage 3 sub strain infections have been associated with different phenotypes for instance, reduced expression of TNFα and IFNγ, reduced growth rate in macrophages [18, 30], causing cavitary TB, pan sensitivity to anti-TB drugs [31] and causing severe disease [18]. Noticeably, TB household population studies can be confounded by a number of factors that could have affected our downward data analysis [32]. Nonetheless, we think our analysis was robust enough since known risk factors, such as patients with a positive smear (OR = 9. 384; CI 95% = 2.603–33.835) were associated with severe disease, HIV reduces (OR = 0.316; CI 95% = 0.114–0.876) the risk of developing severe disease [33, 34]. Additionally, the data showed that patients with BCG scar (OR = 0.295; CI 95% = 0.102–0.854) and swollen lymph nodes (lymphadenitis) were less likely to develop advanced severe disease. Presence of scar on the shoulders suggests that the patients were vaccinated with a BCG vaccine. The efficacy of the BCG vaccine has been found to be variable in conferring protection against *M*. *tuberculosis* infection [35, 36]. For instance BCG vaccination is not protective to *M*. *tuberculosis* Beijing (MTB lineage 2) strains [12, 37], but is protective of lineage 4 (H37RV, Harlem) and *M*. *canetti* strains [38]. This data therefore suggests that BCG vaccination might be protective against the development of advance severe disease in *M*. *tuberculosis* lineage 3 sub strains infections. Whether this is true between lineages, another study can elucidate on this observation. In addition, the data suggests that patients with lymphadenitis (OR = 0.171; CI 95% = 0.034–0.856) are less likely to develop severe disease. This could be for two reasons; perhaps patients had other infections that caused the lymphadenitis and not *M*. *tuberculosis* lineage 3 infections per say. Secondly, trafficking of *M*. *tuberculosis* from the primary foci (most often the lung depending on the route of infection) to the regional lymph nodes causes inflammation and subsequent localization of the bacillus in the lymphatic tissues a scenario referred to as extra pulmonary tuberculosis. Studies have demonstrated that *M*. *tuberculosis* sub lineages preferentially targets pulmonary (lungs) or extra pulmonary tissues (lymph nodes, bones, intestines, meninges among others) [39, 40]. For instance, the Euro American lineage is associated with pulmonary tuberculosis [41], Beijing strains are associated with severe lung pathology [15], the East Africa India strains cause a less severe pulmonary disease [42] and CAS strains are more prevalent in extra pulmonary tuberculosis infections [27, 43].

### Limitations

Because MTB-L3 is not common in Uganda, our analyses of the sub lineages were limited by sample size, resulting in large confidence intervals and a potential loss of statistical power. Secondly, there was a selection bias (index patient) in recruitment of the patients which could inherently skew the findings. Thirdly, the study did not explore the possibilities of other comorbid diseases among the TB patients which could impact our results. Our approach could have been inferior to other genotyping techniques such MIRU-VNTR, whole genome sequencing in resolving sub lineages. However, the strength of this study is that we used a robust SNP typing assay to delineate MTB- main lineages 3, this improves on the accuracy of defining the sub lineages.

### Conclusions

In Kampala, Uganda, there are sub lineages of *M*. *tuberculosis* lineage 3, of which CAS-Dehli is the most predominant. None of these is associated with increased risk of causing severe disease. Patients infected with M. tuberculosis lineage 3 strains who have lymphadenitis or have a BCG scar are less likely to develop severe disease; patients with a positive smear have a higher risk of developing severe disease”

## Supporting information

S1 Table Spoligotype pattern of *M*.*tuberculosis* lineage 3 strains. (DOCX)

S2 Table *M*.*tuberculosis* lineage 3 strains spoligotypes with unknown shared international type numbers (SIT #). (DOCX)

## References

[1] Brites D, Gagneux S. Co-evolution of Mycobacterium tuberculosis and Homo sapiens. Immunological reviews. 2015;264(1):6–24. 10.1111/imr.12264 25703549 PMC4339235

[2] Firdessa R, Berg S, Hailu E, Schelling E, Gumi B, Erenso G, et al Mycobacterial lineages causing pulmonary and extrapulmonary tuberculosis, Ethiopia. Emerging infectious diseases. 2013;19(3):460–3. 10.3201/eid1903.120256 23622814 PMC3647644

[3] Coscolla M, Gagneux S. Consequences of genomic diversity in Mycobacterium tuberculosis. Seminars in immunology. 2014;26(6):431–44. 10.1016/j.smim.2014.09.012 25453224 PMC4314449

[4] Gagneux S, DeRiemer K, Van T, Kato-Maeda M, de Jong BC, Narayanan S, et al Variable host-pathogen compatibility in Mycobacterium tuberculosis. Proceedings of the National Academy of Sciences of the United States of America. 2006;103(8):2869–73. 10.1073/pnas.0511240103 16477032 PMC1413851

[5] Gagneux S, Small PM. Global phylogeography of Mycobacterium tuberculosis and implications for tuberculosis product development. The Lancet Infectious diseases. 2007;7(5):328–37. 10.1016/S1473-3099(07)70108-1 . 17448936

[6] Comas I, Homolka S, Niemann S, Gagneux S. Genotyping of genetically monomorphic bacteria: DNA sequencing in Mycobacterium tuberculosis highlights the limitations of current methodologies. PloS one. 2009;4(11):e7815 10.1371/journal.pone.0007815 19915672 PMC2772813

[7] Wampande EM, Hatzios SK, Achan B, Mupere E, Nsereko M, Mayanja HK, et al A single-nucleotide-polymorphism real-time PCR assay for genotyping of Mycobacterium tuberculosis complex in peri-urban Kampala. BMC infectious diseases. 2015;15:396 10.1186/s12879-015-1121-7 26423522 PMC4590274

[8] Brudey K, Driscoll JR, Rigouts L, Prodinger WM, Gori A, Al-Hajoj SA, et al Mycobacterium tuberculosis complex genetic diversity: mining the fourth international spoligotyping database (SpolDB4) for classification, population genetics and epidemiology. BMC microbiology. 2006;6:23 10.1186/1471-2180-6-23 16519816 PMC1468417

[9] Comas I, Coscolla M, Luo T, Borrell S, Holt KE, Kato-Maeda M, et al Out-of-Africa migration and Neolithic coexpansion of Mycobacterium tuberculosis with modern humans. Nature genetics. 2013;45(10):1176–82. 10.1038/ng.2744 23995134 PMC3800747

[10] Brites D, Gagneux S. Old and new selective pressures on Mycobacterium tuberculosis. Infection, genetics and evolution: journal of molecular epidemiology and evolutionary genetics in infectious diseases. 2012;12(4):678–85. 10.1016/j.meegid.2011.08.010 21867778 PMC3253320

[11] Wampande EM, Mupere E, Debanne SM, Asiimwe BB, Nsereko M, Mayanja H, et al Long-term dominance of Mycobacterium tuberculosis Uganda family in peri-urban Kampala-Uganda is not associated with cavitary disease. BMC infectious diseases. 2013;13:484 10.1186/1471-2334-13-484 24134504 PMC3853102

[12] Parwati I, van Crevel R, van Soolingen D. Possible underlying mechanisms for successful emergence of the Mycobacterium tuberculosis Beijing genotype strains. The Lancet Infectious diseases. 2010;10(2):103–11. 10.1016/S1473-3099(09)70330-5 . 20113979

[13] Ordway D, Henao-Tamayo M, Shanley C, Smith EE, Palanisamy G, Wang B, et al Influence of Mycobacterium bovis BCG vaccination on cellular immune response of guinea pigs challenged with Mycobacterium tuberculosis. Clinical and vaccine immunology: CVI. 2008;15(8):1248–58. 10.1128/CVI.00019-08 18508930 PMC2519313

[14] Ordway DJ, Shang S, Henao-Tamayo M, Obregon-Henao A, Nold L, Caraway M, et al Mycobacterium bovis BCG-mediated protection against W-Beijing strains of Mycobacterium tuberculosis is diminished concomitant with the emergence of regulatory T cells. Clinical and vaccine immunology: CVI. 2011;18(9):1527–35. 10.1128/CVI.05127-11 21795460 PMC3165219

[15] Kato-Maeda M, Shanley CA, Ackart D, Jarlsberg LG, Shang S, Obregon-Henao A, et al Beijing sublineages of Mycobacterium tuberculosis differ in pathogenicity in the guinea pig. Clinical and vaccine immunology: CVI. 2012;19(8):1227–37. 10.1128/CVI.00250-12 22718126 PMC3416080

[16] Coscolla M, Gagneux S. Does M. tuberculosis genomic diversity explain disease diversity? Drug discovery today Disease mechanisms. 2010;7(1):e43–e59. 10.1016/j.ddmec.2010.09.004 21076640 PMC2976975

[17] Lukoye D, Katabazi FA, Musisi K, Kateete DP, Asiimwe BB, Okee M, et al The T2 Mycobacterium tuberculosis genotype, predominant in Kampala, Uganda, shows negative correlation with antituberculosis drug resistance. Antimicrobial agents and chemotherapy. 2014;58(7):3853–9. 10.1128/AAC.02338-13 24777100 PMC4068514

[18] Newton SM, Smith RJ, Wilkinson KA, Nicol MP, Garton NJ, Staples KJ, et al A deletion defining a common Asian lineage of Mycobacterium tuberculosis associates with immune subversion. Proceedings of the National Academy of Sciences of the United States of America. 2006;103(42):15594–8. 10.1073/pnas.0604283103 17028173 PMC1622867

[19] Stucki D, Brites D, Jeljeli L, Coscolla M, Liu Q, Trauner A, et al Mycobacterium tuberculosis lineage 4 comprises globally distributed and geographically restricted sublineages. Nature genetics. 2016;48(12):1535–43. 10.1038/ng.3704 27798628 PMC5238942

[20] Hershberg R. Human host range of Mycobacterium tuberculosis. Nature genetics. 2016;48(12):1453–4. 10.1038/ng.3724 27898082

[21] Stein CM, Zalwango S, Malone LL, Thiel B, Mupere E, Nsereko M, et al Resistance and Susceptibility to Mycobacterium tuberculosis Infection and Disease in Tuberculosis Households in Kampala, Uganda. American journal of epidemiology. 2018;187(7):1477–89. 10.1093/aje/kwx380 29304247 PMC6031055

[22] Kamerbeek J, Schouls L, Kolk A, van Agterveld M, van Soolingen D, Kuijper S, et al Simultaneous detection and strain differentiation of Mycobacterium tuberculosis for diagnosis and epidemiology. Journal of clinical microbiology. 1997;35(4):907–14. 9157152 10.1128/jcm.35.4.907-914.1997 PMC229700

[23] Couvin D, David A, Zozio T, Rastogi N. Macro-geographical specificities of the prevailing tuberculosis epidemic as seen through SITVIT2, an updated version of the Mycobacterium tuberculosis genotyping database. Infection, genetics and evolution: journal of molecular epidemiology and evolutionary genetics in infectious diseases. 2018 10.1016/j.meegid.2018.12.030 . 30593925

[24] Falk AP P.C. Classification of pulmonary tuberculosis Diagnosis standards and classification of tuberculosis. Edited by: Falk A, O'Connor AJB, Pratt PC, Webb JA, Weir JA, Wolinsky A. 1969, New York, NY: National Tuberculosis and Respiratory Disease Association, 12: 68–76.

[25] Asiimwe BB, Ghebremichael S, Kallenius G, Koivula T, Joloba ML. Mycobacterium tuberculosis spoligotypes and drug susceptibility pattern of isolates from tuberculosis patients in peri-urban Kampala, Uganda. BMC infectious diseases. 2008;8:101 10.1186/1471-2334-8-101 PubMed Central PMCID: PMC2519071. 18662405 PMC2519071

[26] Bazira J, Asiimwe BB, Joloba ML, Bwanga F, Matee MI. Mycobacterium tuberculosis spoligotypes and drug susceptibility pattern of isolates from tuberculosis patients in South-Western Uganda. BMC infectious diseases. 2011;11:81 10.1186/1471-2334-11-81 21453482 PMC3100262

[27] Wamala D, Okee M, Kigozi E, Couvin D, Rastogi N, Joloba M, et al Predominance of Uganda genotype of Mycobacterium tuberculosis isolated from Ugandan patients with tuberculous lymphadenitis. BMC research notes. 2015;8:398 10.1186/s13104-015-1362-y 26323435 PMC4556223

[28] Asiimwe BB, Koivula T, Kallenius G, Huard RC, Ghebremichael S, Asiimwe J, et al Mycobacterium tuberculosis Uganda genotype is the predominant cause of TB in Kampala, Uganda. The international journal of tuberculosis and lung disease: the official journal of the International Union against Tuberculosis and Lung Disease. 2008;12(4):386–91. . 18371263

[29] Dickman KR, Nabyonga L, Kateete DP, Katabazi FA, Asiimwe BB, Mayanja HK, et al Detection of multiple strains of Mycobacterium tuberculosis using MIRU-VNTR in patients with pulmonary tuberculosis in Kampala, Uganda. BMC infectious diseases. 2010;10:349 10.1186/1471-2334-10-349 21143966 PMC3004912

[30] Tanveer M, Hasan Z, Kanji A, Hussain R, Hasan R. Reduced TNF-alpha and IFN-gamma responses to Central Asian strain 1 and Beijing isolates of Mycobacterium tuberculosis in comparison with H37Rv strain. Transactions of the Royal Society of Tropical Medicine and Hygiene. 2009;103(6):581–7. 10.1016/j.trstmh.2009.03.014 . 19375139

[31] Chatterjee A, D'Souza D, Vira T, Bamne A, Ambe GT, Nicol MP, et al Strains of Mycobacterium tuberculosis from western Maharashtra, India, exhibit a high degree of diversity and strain-specific associations with drug resistance, cavitary disease, and treatment failure. Journal of clinical microbiology. 2010;48(10):3593–9. 10.1128/JCM.00430-10 20720028 PMC2953068

[32] Malik AN, Godfrey-Faussett P. Effects of genetic variability of Mycobacterium tuberculosis strains on the presentation of disease. The Lancet Infectious diseases. 2005;5(3):174–83. 10.1016/S1473-3099(05)01310-1 . 15766652

[33] Aderaye G, Bruchfeld J, Assefa G, Feleke D, Kallenius G, Baat M, et al The relationship between disease pattern and disease burden by chest radiography, M. tuberculosis Load, and HIV status in patients with pulmonary tuberculosis in Addis Ababa. Infection. 2004;32(6):333–8. 10.1007/s15010-004-3089-x . 15597222

[34] Altet-Gomez MN, Alcaide J, Godoy P, Romero MA, Hernandez del Rey I. Clinical and epidemiological aspects of smoking and tuberculosis: a study of 13,038 cases. The international journal of tuberculosis and lung disease: the official journal of the International Union against Tuberculosis and Lung Disease. 2005;9(4):430–6. . 15830749

[35] Mangtani P, Abubakar I, Ariti C, Beynon R, Pimpin L, Fine PE, et al Protection by BCG vaccine against tuberculosis: a systematic review of randomized controlled trials. Clinical infectious diseases: an official publication of the Infectious Diseases Society of America. 2014;58(4):470–80. 10.1093/cid/cit790 . 24336911

[36] Roy A, Eisenhut M, Harris RJ, Rodrigues LC, Sridhar S, Habermann S, et al Effect of BCG vaccination against Mycobacterium tuberculosis infection in children: systematic review and meta-analysis. Bmj. 2014;349:g4643 10.1136/bmj.g4643 25097193 PMC4122754

[37] van Soolingen D, Qian L, de Haas PE, Douglas JT, Traore H, Portaels F, et al Predominance of a single genotype of Mycobacterium tuberculosis in countries of east Asia. Journal of clinical microbiology. 1995;33(12):3234–8. 8586708 10.1128/jcm.33.12.3234-3238.1995 PMC228679

[38] Lopez B, Aguilar D, Orozco H, Burger M, Espitia C, Ritacco V, et al A marked difference in pathogenesis and immune response induced by different Mycobacterium tuberculosis genotypes. Clinical and experimental immunology. 2003;133(1):30–7. 10.1046/j.1365-2249.2003.02171.x 12823275 PMC1808750

[39] Click ES, Moonan PK, Winston CA, Cowan LS, Oeltmann JE. Relationship between Mycobacterium tuberculosis phylogenetic lineage and clinical site of tuberculosis. Clinical infectious diseases: an official publication of the Infectious Diseases Society of America. 2012;54(2):211–9. 10.1093/cid/cir788 . 22198989

[40] Srilohasin P, Chaiprasert A, Tokunaga K, Nishida N, Prammananan T, Smittipat N, et al Genetic diversity and dynamic distribution of Mycobacterium tuberculosis isolates causing pulmonary and extrapulmonary tuberculosis in Thailand. Journal of clinical microbiology. 2014;52(12):4267–74. 10.1128/JCM.01467-14 25297330 PMC4313297

[41] Caws M, Thwaites G, Dunstan S, Hawn TR, Lan NT, Thuong NT, et al The influence of host and bacterial genotype on the development of disseminated disease with Mycobacterium tuberculosis. PLoS pathogens. 2008;4(3):e1000034 10.1371/journal.ppat.1000034 18369480 PMC2268004

[42] Albanna AS, Reed MB, Kotar KV, Fallow A, McIntosh FA, Behr MA, et al Reduced transmissibility of East African Indian strains of Mycobacterium tuberculosis. PloS one. 2011;6(9):e25075 10.1371/journal.pone.0025075 21949856 PMC3176299

[43] Sankar MM, Singh J, Diana SC, Singh S. Molecular characterization of Mycobacterium tuberculosis isolates from North Indian patients with extrapulmonary tuberculosis. Tuberculosis. 2013;93(1):75–83. 10.1016/j.tube.2012.10.005 . 23140853

