# Supplementary material for: Genetic variability and consequence of Mycobacterium tuberculosis lineage 3 in Kampala-Uganda
Source: PLoS One. 2019 Sep 9;14(9):e0221644. doi: 10.1371/journal.pone.0221644 (PMC6733460; doi:10.1371/journal.pone.0221644)
Supplement: S1 Table — (DOCX) [file pone.0221644.s001.docx]

# Supporting information

**S 1 Table**

| **SIT #** | **Frequency** | **Sub lineage** | **Spoligotype pattern** |
| --- | --- | --- | --- |
| 26 | 16 | CAS-DEHLI | ■■■□□□□■■■■■■■■■■■■■■■□□□□□□□□□□□□■■□□■■■■■ |
| 289 | 2 | CAS-DEHLI | ■■■□□□□■■■■■■■■■■■■■■■□□□□□□□□□□□□■■■□■■■■■ |
| 1198 | 7 | CAS-DEHLI | ■■■□□□□■■■■■□■■■■■■■■■□□□□□□□□□□□□■■□□■■■■■ |
| **1314** | **4** | CAS-DEHLI | ■■■□□□□■■□■■■■■■■■■■■■□□□□□□□□□□□□■■□□■■■■■ |
| **2145** | **2** | CAS-DEHLI | ■■■□□□□■■■■■■■■■■■■■□■□□□□□□□□□□□□■■■■■■■■■ |
| **2359** | **3** | CAS-DEHLI | ■■■□□□□■■■■□■■■■■■■■■■□□□□□□□□□□□□■■□□■■■■■ |
| **26** | **43** | CAS-DEHLI | ■■■□□□□■■■■■■■■■■■■■■■□□□□□□□□□□□□■■■■■■■■■ |
| **21** | **22** | **CAS-KILLI** | ■■■□□□□■■□■■■■■■■■■□□□□□□□□□□□□□□□□■■■■■■■■ |
| **288** | **10** | **CAS 2** | ■■■□□□□□□□■■■■■■■■■■■■□□□□□□□□□□□□■■■■■■■■■ |
| **142** | **2** | **CAS** | ■■■□□□□■■■■■■■■■■■■■■□□□□□□□□□□□□□■■■■■■■■■ |
| **Orphan** | **17** | **Orphan** | **See supplementary Table 2 (S 2 Table)** |
| **523** | **2** | **Manu-ancestor** | ■■■■■■■■■■■■■■■■■■■■■■■■■■■■■■■■■■■■■■■■■■■ |
| **1089** | **2** | **Unknown** | ■□□□□□□■■■■■■■■■■■■■■■□□□□□□□□□□□□■■■■■■■■■ |
| **266** | **9** | **Atypic** | □□□□□□□□□□□□□□□□□□□□□□□□□□□□□□□□□□□□□□□□□□□ |
