# Supplementary material for: Genetic variability and consequence of Mycobacterium tuberculosis lineage 3 in Kampala-Uganda
Source: PLoS One. 2019 Sep 9;14(9):e0221644. doi: 10.1371/journal.pone.0221644 (PMC6733460; doi:10.1371/journal.pone.0221644)
Supplement: S2 Table — (DOCX) [file pone.0221644.s002.docx]

**S2 Table**

| **SIT #** | **Frequency** | **Spoligotype pattern** | **Sub lineage** |
| --- | --- | --- | --- |
| **Orphan** | **1** | \| ■■■■■■■■■■■■■■■■■■■■■■□□■■□□□□■■□□■■■■■■■■■ \| \| --- \| | **Unknown** |
| **Orphan** | **1** | \| ■■■■■■■■■□■■■■■■■■■■■■■■■■■■■■■■□□■■■■■■■■■ \| \| --- \| | **Unknown** |
| **Orphan** | **1** | \| ■■■□■■■□■■■■■■■■■■■■□□□□■■□□□□■■□□□□■■■■■■■ \| \| --- \| | **Unknown** |
| **Orphan** | **1** | \| ■■■□□■□■■□■■■■■■■■■■■■■■■■□□■□■■□□■■■■■■■■■ \| \| --- \| | **Unknown** |
| **Orphan** | **1** | \| ■■■□□□□■■■■■■■■■■■■□■■□□□□□□□□□□□□□□□□■■■■■ \| \| --- \| | **Unknown** |
| **Orphan** | **1** | \| ■■■□□□□■■■■■□□■■■■■■■■□□□□□□□□□□□□■■□■■□■■■ \| \| --- \| | **Unknown** |
| **Orphan** | **1** | \| ■■■□□□□■■□■■■■■■■■■□□□□□□□□□□□□□□□□■■□□■■■■ \| \| --- \| | **Unknown** |
| **Orphan** | **1** | \| ■■■□□□□■■□■■■■□■■■■□□□□□□□□□□□□□□□■■■■□■■■■ \| \| --- \| | **Unknown** |
| **Orphan** | **2** | \| ■■■□□□□■■□■■■□■■■■■■■■□□□□□□□□□□□□■■■■■■■■■ \| \| --- \| | **Unknown** |
| **Orphan** | **1** | \| ■■■□□□□□□■■■■■■■■■■■□□□□□□□□□□□□□□□■■■■■■■■ \| \| --- \| | **Unknown** |
| **Orphan** | **1** | \| ■■■□□□□□□□■■■■■■■■■■■■□□□■■■■■■■□□■■■■■■■■■ \| \| --- \| | **Unknown** |
| **Orphan** | **1** | \| ■■■□□□□□□□■■■□■■■■■■■■□□□□□□□□□□□□■■■■■■■■■ \| \| --- \| | **Unknown** |
| **Orphan** | **1** | \| ■□■□□□□□■□■□■□□■■■■■■□□□□□□□□□□□□□■■□■□■■□■ \| \| --- \| | **Unknown** |
| **Orphan** | **2** | \| □□□□□□□■■■■■■■■■■■■■■■■■■■■■■■■■□□□□■■■□■■■ \| \| --- \| | **Unknown** |
| **Orphan** | **1** | \| ■■■□□□□■■■■■■■■■■■■□■■□□□□□□□□□□□□□□□□■■■■■ \| \| --- \| | **Unknown** |
